# Supplementary material for: Mean Velocity of the Pulmonary Artery as a Clinically Relevant Prognostic Indicator in Patients with Heart Failure with Preserved Ejection Fraction
Source: J Clin Med. 2022 Jan 19;11(3):491. doi: 10.3390/jcm11030491 (PMC8836987; doi:10.3390/jcm11030491)
Supplement: Supplementary file 1 [file jcm-11-00491-s001.zip › jcm-1509276-SI.pdf]

Supplementary Table S1. Baseline clinical and imaging characteristics according to the performance of right heart catheterization.

	No RHC (n=30)	RHC (n=28)	Total sample (n=58)	p-value
Age (years)	68.7±13.9	66±13.2	67.5±13.5	0.454
Sex, male (n,%)	21(70)	13(46.4)	34(58.6)	0.069
BSA (m^2)	1.8±0.13	1.7±0.18	1.8±0.17	0.010
Arterial hypertension (n,%)	13(43.3)	17(60.7)	30(51.7)	0.186
Diabetes mellitus (n,%)	5(16.7)	5(17.9)	10(17.2)	0.905
Dislipidemia (n,%)	13(43.3)	11(39.3)	24(41.4)	0.754
Atrial fibrillation (n,%)	12(40)	15(53.6)	27(46.6)	0.300
LBBB	4(13.3)	1(3.6)	5(8.6)	0.186
Previous coronary artery disease (n,%)	6(20)	2(7.1)	8(13.8)	0.156
Glomerular filtration rate (ml/min/1.73m^2)	78.3±15.3	69.4±19.7	72.9±17.8	0.151
Stage 3-4 chronic kidney disease (n,%)	3(10)	3(10.7)	6(10.3)	0.962
NT-proBNP (pg/mL)	1032.2±991.5	1349.9±1241.9	1281.8±1164.5	0.692
Prior HF hospitalization (n,%)	4(13.3)	8(28.6)	12(20.7)	0.152
NYHA functional class (n,%)<br>I<br>II<br>III<br>IV	16(53.3)<br>13(43.3)<br>0<br>1(3.3)	3(10.7)<br>11(39.3)<br>10(35.7)<br>4(14.3)	19(32.8)<br>24(41.4)<br>10(17.2)<br>5(8.6)	<0.001
NYHA III-IV/IV (n,%)	1(3.3)	14(50)	15(25.9)	<0.001
Cerebrovascular disease (n,%)	3(10)	5(17.9)	8(13.8)	0.386
**Echocardiography-parameters**				
LVEF (%)	56.5±8.7	60.2±9.3	58.3±9.1	0.134
LV septum width (mm)	12.7±1.8	12.2±1.6	12.4±1.7	0.367
LV posterior wall width (mm)	10.9±1.9	11.9±1.9	11.1±1.9	0.553
LVEDD (mm)	47.5±6.6	47.1±6.8	47.2±6.6	0.818
LVESD (mm)	34.7±7.4	30±12.9	32.5±10.5	0.201
Indexed left atrial volume (ml/m^2)	43.9±19.8	50.8±16.5	47.1±18.5	0.204
E/A ratio	0.9±0.7	1.3±0.6	1.2±0.7	0.167
DT (ms)	218.6±49.9	195.5±44.6	208.4±48.1	0.241
e´ (septal)	6.2±1.8	6.8±1.6	6.5±1.9	0.326
e´ (lateral)	11.1±4.5	9.5±2.5	10.2±3.5	0.218
E/e´ ratio (lateral)	7.2±3.6	8.0±2.7	7.5±3.3	0.493
TAPSE (mm)	22.1±4.9	19.6±3.9	20.9±4.6	0.052
S´ tricuspid (cm/s)	12.3±2.9	9.5±2.6	10.9±3	0.009
Pulmonary acceleration time (ms)	96.3±21.6	76.7±22.8	85.9±24	0.015
PAPs (mmHg)	39.6±12.1	51.2±17.6	45.6±16.1	0.010
TAPSE/PAPs	0.59±0.2	0.42±0.2	0.5±0.2	0.008
TR grade ≥ 3/4	3(10)	8(28.6)	11(19)	0.071
**CMR-parameters**				
LVEF (%)	58.6±8.4	60.5±8.9	59.5±8.7	0.400
iLVEDV (ml/m^2)	86±28.6	75.8±25.5	81.1±27.4	0.158
iLVESV (ml/m^2)	38.2±19.9	32±20.3	35.2±20.1	0.247
Left ventricular mass (gr)	77.5±24.4	66.5±19.4	72±22.5	0.068
RVEF (%)	56.3±9.5	52.5±12	54.4±10.9	0.197
iRVEDV (ml/m^2)	95.3±33.7	101.8±28.5	98.5±31.1	0.437
iRVESV (ml/m^2)	43.3±23.9	47.9±16.8	45.6±20.7	0.408
LGE (n,%)	8(26.7)	15(53.6)	23(39.7)	0.041

LGE ischemic pattern (n,%)	5(16.7)	15(53.6)	5(8.6)	0.004
LGE non-ischemic pattern (n,%)	4(13.3)	1(3.6)	20(34.5)	0.173
Left atrial area (mm^2)	15.7±4.9	18.2±4.3	16.9±4.7	0.061
Right atrial area (mm^2)	15.1±5.6	18.7±8.9	16.8±7.5	0.095
Maximal PA area (cm^2)	9.1±3.4	9.9±3	9.5±3.2	0.354
Minimal PA area (cm^2)	7.1±2.7	8.1±2.5	7.6±2.6	0.168
PA pulsatility (%)	29.3±16.5	23.7±15.4	26.7±16.1	0.194
Right ventricular Ea/Emax	0.83±0.37	1.02±0.62	0.93±0.51	0.152
mvPA (cm/s)	10.9±3.9	8.6±3.6	9.8±3.9	0.022
PVR-CMR (Wood Units)	4±1.8	5.6±2.5	4.8±2.3	0.008
**Cardiovascular events**				
Readmission for decompensated heart failure (n,%)	5(16.7)	10(35.7)	15(25.9)	0.098
All-cause death (n,%)	6(20)	2(7.1)	8(13.8)	0.156
Primary combined endpoint (n,%)	9(30)	12(42.9)	21(36.2)	0.309

BSA: body surface area. HF: heart failure. LBBB: left bundle branch block. mvPA: mean velocity pulmonary artery.

Supplemental Table S2. Baseline clinical characteristics according to mvPA.

	**mvAP ≤ 9cm/s (n=30)**	**mvAP > 9cm/s (n=28)**	**Total sample (n=58)**	**p-value**
Age (years)	67.7±11.9	67.3±15.3	67.5±13.5	0.821
Sex, male (n,%)	15(50)	19(67.9)	34(58.6)	0.168
BSA (m^2)	1.76±0.17	1.81±0.17	1.8±0.17	0.077
Arterial hypertension (n,%)	17(56.7)	13(46.4)	30(51.7)	0.436
Diabetes mellitus (n,%)	7(23.3)	3(10.7)	10(17.2)	0.204
Dislipidemia (n,%)	14(46.7)	10(35.7)	24(41.4)	0.397
Atrial fibrillation (n,%)	14(46.7)	13(46.4)	27(46.6)	0.986
LBBB	3(10)	2(7.1)	5(8.6)	0.698
Previous coronary artery disease (n,%)	3(10)	5(17.9)	8(13.8)	0.386
Glomerular filtration rate (ml/min/1.73m^2)	75.9±16.5	69.3±18.9	72.9±17.8	0.250
Stage 3-4 chronic kidney disease (n,%)	1(3.4)	5(17.9)	6(10.3)	0.061
NT-proBNP (pg/mL)	1469.6±1443.5	1094±878.6	1281.8±1164.5	0.456
Prior HF hospitalization (n,%)	6(20)	6(21.4)	12(20.7)	0.893
NYHA functional class (n,%)				0.224
I	8(26.7)	11(39.3)	19(32.8)	
II	11(36.7)	13 (46.4)	24(41.4)	
III	8(26.7)	2(7.1)	10(17.2)	
IV	3(10)	2(7.1)	5(8.6)	
NYHA III-IV/IV (n, %)	4(13.3)	4(14.3)	8(13.8)	0.916
Cerebrovascular disease (n,%)	67.7±11.9	67.3±15.3	67.5±13.5	0.821

BSA: body surface area. HF: heart failure. LBBB: left bundle branch block. mvPA: mean velocity pulmonary artery.
